# Turnover intention and related factors among general practitioners in Hubei, China: a cross-sectional study

**DOI:** 10.1186/s12875-018-0752-3

**Published:** 2018-05-24

**Authors:** Yong Gan, Yanhong Gong, Yawen Chen, Shiyi Cao, Liqing Li, Yanfeng Zhou, Chulani Herath, Wenzhen Li, Xingyue Song, Jing Li, Tingting Yang, Xiaoxv Yin, Zuxun Lu

**Affiliations:** 1 Department of Social Medicine and Health Management, School of Public Health, Tongji Medical College, Huazhong University of Science and Technology, No. 13 Hangkong Road, Wuhan, 430030 Hubei China; 2 Department of Management, School of Economics and Management, Jiangxi Science and Technology Normal University, Nanchang, Jiangxi China

**Keywords:** Personnel turnover, Primary care, Epidemiology, General practitioners, China

## Abstract

**Background:**

High turnover among general practitioners (GPs) is a significant challenge in China’s efforts to build a sustainable, effective primary care system, but little data is available to help understand and address this issue. The study was aiming at assessing the intention to leave their posts among a sample of GPs and investigating associated factors.

**Methods:**

A cross-sectional survey was conducted between December 12, 2014 and March 10, 2015 in Hubei Province, Central China. A total of 1016 GPs (response rate, 85.67%) were investigated by using a structured self-administered questionnaire. A generalized linear regression model was used to identify the associated factors with turnover intention among GPs.

**Results:**

Based on a full score of 24, the average score for GPs’ turnover intention was 15.40 (SD = 3.43). 78.35% of the GPs had a moderate or higher level of turnover intention. Six hundred and thirty one (62.37%) GPs had ever been exposed to abuse of any kind (physical assault, 18.92%; verbal abuse, 54.38%; threat, 33.79%; verbal sexual harassment, 22.66%; and physical sexual harassment, 7.59%). Generalized linear regression analysis indicated that GPs who were male; who had a vocational school or higher; who had a temporary work contract; who were with lower level of job satisfaction; who reported higher scores on emotional exhaustion; who had been exposed to higher frequency of workplace violence were expressed higher intention to leave their present positions.

**Conclusion:**

This study shows that GP’s intention to leave general practices is high in Hubei, China. In addition, the prevalence of workplace violence is high among GPs, particularly in the verbal abuse and threat. Measures such as offering permanent contract status, increasing overall job satisfaction, and improving doctor-patient relationship, are needed to moderate GP’s turnover intention in order to maintain the foundation of China’s three-tier health system.

**Electronic supplementary material:**

The online version of this article (10.1186/s12875-018-0752-3) contains supplementary material, which is available to authorized users.

## Background

General practitioners (GPs) are the foundation of any primary healthcare system. They are the main providers of community health service (CHS), providing a wide range of health services and managing referrals to specialist care and hospitals. Their quality and quantity are directly associated with the effectiveness and quality of the health services of a nation. Such GPs system is widely established in western countries, such as the United States, the United Kingdom (UK), Germany, and Australia.

The latest round of nationwide systemic reform in China was launched in 2009, which aimed to provide affordable, equitable access to quality basic health care for all its citizens by 2020. The building and strengthening of the infrastructure for primary healthcare was the core task of the reform, supported by infusions of substantial public funding [1]. In 2011, the instructional advice on establishment of GPs system was announced through the Stated Council of China, and it proposed to train qualified and excellent GPs by a variety of ways [2]. In 2015, the Stated Council of China pointed out that strengthening of the infrastructure for primary healthcare was the importance of establishing the grading healthcare system, which needed to promote the gatekeeper role of GPs [3]. Many efforts were taken to strengthen the establishment and expand the supply of primary health workers, especially in the training and recruitment of GPs [4].Primary healthcare workers has achieved the attention and support from the government, leaders and related scholars, and the building of GPs team made an some great progress, however, in China, primary health institutions (community health centers (CHCs) and township health centers (THCs)) have been facing ongoing challenges in the shortage of human resources and turnover of on-post GPs. According to the China Health Statistics Yearbook in 2013, the proportion of GPs among registered physicians was 4.2% [5], which was lower than the level ranging from 30% to 60% of western countries [6, 7]. In 2014, the government has identified training GPs as a top priority and planned to train 300,000 of them by 2020, an ambitious goal by itself. Retention of qualified health professionals in primary healthcare institutions, especially in poor regions, is even more difficult [4]. According to the development planning of Chinese healthcare systems [8], in 2020, two to three GPs per 10,000 population will be fitted in the healthcare system in providing care to people at different levels in China.

Turnover, a behavior of actually leaving, was an important value in human resources management and maintenance of the current workforce [9].Intention to leave was a future behavior of actually leaving a profession or an organization [10], which was considered as a proxy of turnover [11]. Wide agreement on core of turnover models was developed in westerns countries that dissatisfaction ultimately causes employee turnover [12–16]. Recently, newly proposed models are often an extension or refinement of these models, which were considered as the core/backbone of the contemporary turnover theory [17]. According to the above mentioned models, job satisfaction and organizational commitment have become the mainstay of turnover theory, and they provided the supports to the hypothesis that turnover was preceded by turnover intention, which was preceded by job satisfaction. In addition, many turnover researchers showed that turnover intention is also influenced by other factors, such as burnout [18, 19] and workplace violence [20–22]. Thus, this study put forward the hypothesis that burnout, job satisfaction, and workplace violence were preceded by turnover intention.

Several studies have been conducted to investigate turnover and its influencing factors (such as socio-demographic, work stress, job satisfaction and burnout) among GPs in developed countries [23–28]. However, there has been almost no data on the turnover of GPs in China now, owing mostly to the difficulty in data collection. Studies on turnover intention, although indirectly, can offer insight on the issue. Some studies have shown that intention to leave general practices could actually translate into GPs leaving their jobs [10, 29, 30]. Moreover, turnover intention may be indicative of lower GPs work morale [29], absenteeism and lowered performance [31], and help developing preemptive measures to stop the turnover before it occurs. According to the background of healthcare reform strengthening the primary health institutions, if the turnover intention was high among Chinese GPs, it would aggravate the shortage of healthcare human resource, lead to decrease productivity, and affect health services quality. This finally could contribute to negative effect on the China’s health-care system reform performance. Thus, assessing the turnover intention among GPs is of critical importance to understand the issue and develop targeted policies to promote the retention of current general practice staff and improvement of primary healthcare in Chinese general practice. To our knowledge, no studies to date have investigated the turnover intention and relevant determinants among GPs in China. This study was to investigate the turnover intention and its influencing factors among GPs who served China’s grassroots communities. In light of Chinese health care reforms, the findings of this study would inform policymakers about priority areas to strengthen the primary care institutions in China.

## Methods

### Ethics statement

The study protocol was approved by the institutional review boards of Tongji Medical College, Huazhong University of Science and Technology, Wuhan, China. All participants read a statement that explained the purpose of the survey and provided written informed consent before participation in the study.

### Study population and sampling

A cross-sectional study was conducted between December 12, 2014 and March 10, 2015 in Wuhan, Hubei Province (Central China).These GPs worked in CHCs in urban and THCs in rural areas. Initially, we investigated the situation of turnover intention by interviewing related health managers and leader of primary healthcare institutions in Hubei before designing the questionnaire. The prevalence of turnover intention ranged from 16% to 38%.Finally the prevalence of turnover intention (π) was considered as 0.30 in this study. According to the formula of random sampling formula, *α* was equal to 0.05, *μ*_*α*/2_*was equal to* 1.96, and *δ* was equal to 3%. Thus, it needed an ideal sample size of 896 according to $\frac{\mu_{\alpha /2}^2\times \pi \times \left(1-\pi \right)}{\delta^2}$.

A stratified sample design was performed in the study. Firstly, in 2012, there were 2325 primary health institutions (CHCs and THCs) in 17 prefecture-level cities of Hubei province, all of which were recruited in this study. Secondly, according to the number of GPs and scales of primary health institutions in Hubei Province, GPs were randomly selected from each primary health institution. Of 3752 eligible GPs, 1186 were randomly selected to attend the health service quality training meeting conducted by Hubei provincial general practice training center, and then these GPs were asked to complete the self-reported questionnaire. A total of 1119 (94.35%) of selected GPs responded to the survey. All together 103 questionnaires were discarded because of too many missing data or logical errors. Finally, 1016 eligible questionnaires remained, giving a response rate of 85.67%.

### Measures

Dependent variable was the turnover intention of GPs. Turnover intention was measured with a modified version of a turnover intention assessment scale, which consisted of 6 items. The Chinese version of these 6 items was modified according to the studies by Mickael and Spector [32] and Li et al. [33]. The items were rated with a four-point Likert scale, ranging from 1 (never, or strongly impossible) to 4 (always, or very possible) (Table 1).The total score of six items was computed as the score for turnover intention, ranging from 6, the minimal intention, to 24, the absolute intention to leave one’s job. The scores of 0–6, 7–12, 13–18, and 19–24 were assigned for the low, moderate, moderate to high and high degree of turnover intention, respectively [33]. In this study, the Cronbach’s alpha coefficient for the scale was 0.74, indicating a good internal consistency. To reduce information bias, all missing values of turnover intention were replaced with the mean of given participants’ responses to other scale items [34]. However, there were still a few missing data among the categorized variables that not filled in, such as age, sex, education level and so on. In addition, we deleted the questionnaires due to logical errors. The logical errors identified as some mismatched socio-demographic characteristics for individuals. For example, a GP reported that age was 30 years old; however, work tenure was more than 15 years. Thus, these questionnaires with the obvious logical errors were discarded.

**Table 1:** Turnover Intention Scale

Items	Often/Strongly possible	Sometimes/Possible	Scarcely/Impossible	Never/Strongly impossible
1. Do you want to leave your present job?				
2. Do you want to look for other similar job?				
3. Do you want to look for other different job?				
4. According to your present status, how much do you feel the possibility to find the suitable post from other institutions?				
5. If you know that there is a suitable post for you in another institution, how much is the possibility for you to achieve the job?				
6. Will you to quit your present post?				

The independent variables covered socio-demographics characteristics (sex, age, marital status), socio-economic status (education level, average monthly income), and work-related factors (practice setting, work tenure, job title, manager responsibility, contract status, burnout, job satisfaction, and workplace violence). The 22-item Maslach Burnout Inventory (MBI-HSS) [35] was used to assess burnout in three subscales emotional exhaustion (EE, 9 items), depersonalization (DP, 5 items), and personal achievement (PA, 8 items), and respondents rated each item on a seven point Likert scale, ranging from 0 (never) to 6 (every day). Higher scores on the EE and DP subscales correspond with higher degrees of burnout, while PA is inversely correlated with burnout. Summary scores for the subscales were achieved by adding the individual item scores and inputting mean values for missing scores if a maximum of two items were missing for EE and PA and one item was missing for DP. The Cronbach’s alphas for the MBI-HSS, EE, DP, and PA were 0.85, 0.91, 0.87 and 0.90, respectively.

Job satisfaction reported by Shi et al. was measured with 11 items using a five-point ordinal scale ranging from 1 (very dissatisfied) to 5 (very satisfied), including three dimensions: satisfaction on relationship (4 items), satisfaction on personal development (3 items) and satisfaction on basic demand (4 items). This is a reliable and valid instrument for measuring job satisfaction among Chinese primary care providers [36]. The score of job satisfaction ranges from 11 to 55. In this study, the Cronbach’s alpha for the job satisfaction was 0.88.

The Chinese version of Workplace Violence Scale (WVS), developed by Wang et al. [37], was with a good reliability and validity instrument for measuring the incidence of workplace violence when applied to medical staff in China. It included 5 items and was measured with a four-point ordinal scale ranging from 0 (never) to 3 (more than 3 times/year) [38, 39]. The score of workplace violence was ranging from 0 to 15. The higher the score was, the more frequent the workplace violence occurred. Additionally, we reported the prevalence of five types of workplace violence. In this study, the Cronbach’s alpha for the workplace violence was 0.82.

### Statistical analysis

Descriptive analysis was conducted on socio-demographics characteristics, socioeconomic factors, and work-related factors. *T* test and one-way analysis of variance (ANOVAs) were used to compare the difference in turnover intention among groups. The turnover intention was abnormal (W = 0.982843, *P* < 0.0001) and there was no clustering within general practices (intra-class correlation =0.02, *P* = 0.0869), thus, a generalized linear model was conducted to examine the associations between independent variables and GPs’ turnover intention.

Turnover intention, as the dependent variable, was treated as continuous variable. In the multivariable model, independent variables included: age (year in continuous), gender (female and male), marital status (unmarried or widow or divorced, married), education level (high school or below, vocational school, bachelor degree or higher), work tenure (year in continuous), average monthly income (< 2500, 2500~, 3500+ RMB Yuan), contract status (permanent, temporary), job title(elementary or less, intermediate or higher), manager responsibility (yes, no), practice setting (urban CHC, rural THC), overall job satisfaction (continuous), EE level (score in continuous), DP level (score in continuous), PA level (score in continuous) and workplace violence (score in continuous. Multicolinearity was assessed using the variance inflation factor [40]. All the statistical analyses were performed using Statistical Package for Social Sciences (SPSS, Inc., Chicago, III, Version 13.0), and all tests were two sided with a significance level of 0.05.

## Results

The main characteristics of the GP participants are reported in Table 2. Of the total of 1, 016 participants (response rate, 85.66%), the mean age was 38.70 (standard deviation (SD) =6.74) years. Most GPs (93.31%) were married, more than half were male, and half attended bachelor degree or above. Of the participants, the mean duration of healthcare practice was 16.33 (SD = 7.90) years, and 34.12% had management responsibility; 33.73% GPs had income per month less than 2500 RMB Yuan. One quarter had a temporary work contract, and more than half located in urban CHCs practices. The mean score for the workplace violence was 2.33 (SD = 3.05). Most GPs (62.37%) had ever been exposed to workplace violence in the 12-month period. The prevalence of physical assault, verbal abuse, threat, verbal sexual harassment, and physical sexual harassment was 18.92%, 54.38%, 33.79%, 22.66% and 7.59%, respectively (Additional file 1: Table 1).

**Table 2:** Descriptive statistics for determinates and associations with turnover intention

		Turnover intention		
Characteristics	*N (%)*	*(SD)*	*t/F*	*P value*
Age (years)^a^				
< 30	84 (8.28)	15.51(2.94)	6.94	0.0001
30~	405 (39.90)	15.79 (3.35)		
40~	475 (46.80)	15.23(3.45)		
50~	51 (5.02)	13.61(4.00)		
Gender^a^				
Male	659 (64.93)	15.75 (3.50)	4.49	< 0.0001
Female	356 (35.07)	14.74 (3.21)		
Education level^a^				
High school or below	86 (8.59)	13.72 (4.00)	17.68	< 0.0001
Vocational school	394 (39.36)	15.15 (3.38)		
Bachelor degree or higher	521 (52.05)	15.91 (3.25)		
Marital status^a^				
Unmarried/widow/divorced	67 (6.69)	15.79 (3.24)	1.00	0.3183
Married	934 (93.31)	15.36 (3.45)		
Work tenure (years)^a^				
< 10	236 (23.30)	15.71 (3.19)	2.06	0.1275
10~	385 (38.00)	15.44 (3.40)		
20~	392 (38.70)	15.15 (3.57)		
Contract status^a^				
Permanent	734 (73.11)	15.27 (3.47)	−2.24	0.0254
Temporary	270 (26.89)	15.81 (3.30)		
Average monthly income(RMB Yuan)^a^				
< 2500	340 (33.73)	15.72 (3.48)	2.62	0.0733
2500~	474 (47.02)	15.16 (3.39)		
3500+	194 (19.25)	15.40 (3.38)		
Job title*				
Elementary or less	482 (47.91)	15.16 (3.41)	−2.11	0.0349
Intermediate or higher	524 (52.09)	15.62 (3.44)		
Manager responsibility^a^				
Yes	333 (34.12)	15.74 (3.45)	1.92	0.0555
No	643 (65.88)	15.30 (3.41)		
Practice setting				0.0065
CHC	563 (55.41)	15.14 (3.35)	−2.73	
THC	453 (44.59)	15.72 (3.50)		
	Mean ± SD			
Overall job satisfaction	34.25 (6.36)	–	–	–
Emotion exhaustion	18.70 (12.05)	–	–	–
Depersonalization	3.89 (4.75)	–	–	–
Reduced personal accomplishment	13.55 (11.78)	–	–	–
Workplace violence^a^	2.33 (3.05)	–	–	–

Abbreviations: *CHC* community health center, *THC* township health center

^a^Percentage of participant with missing data on age (0.10%), gender (0.10%), education level (1.48%), marital status (1.48%), work tenure (0.30%), contract status (1.18%), average monthly income (0.79%), job title (0.98%), manager responsibility (3.94%), and workplace violence (0.10%)

The mean score of the turnover intention was 15.40 (SD = 3.43), indicating that the GPs generally had a moderate level of turnover intention. In this study, the percent of GPs with a low, moderate, moderate or higher level turnover intention was 0.98%, 20.67%, and 78.35% respectively. *T* tests and ANOVA analysis were conducted to compare the difference in mean scores of turnover intention among groups, and the results are shown in Table 2. There were significant differences in GPs’ turnover intention in terms of age, sex, education level, contract status, job title, practice setting, job satisfaction, burnout, and workplace violence (*P* < 0.05). There were no significant differences in turnover intention among GPs in terms of marital status, work tenure, average monthly income and manager responsibility.

The results of the generalized linear model to identify factors associated with GPs’ turnover intention are shown in Table 3. Gender, education level, contract status, overall job satisfaction, EE level, and exposure to workplace violence were significantly associated with the turnover intention. Those GPs who were male; who had a vocational school or higher; who had a temporary work contract; who were with lower level of job satisfaction; who reported higher scores on the EE; who had been exposed to higher frequency of workplace violence were more likely to show the intention to leave their present position. The adjusted variance in turnover intention explained by the exposure variables was 28%. The explained variance of this model (R^2^) was 0.28. Multicolinearity was not founded since the variance inflation factor of these variables was well below the traditional cut-off value of 10. No interaction of variables was found.

**Table 3:** General linear model analysis for the association with the turnover intention among GP (*N* = 922)

Variables	Estimate	*SE*	*t*	*P value*
Intercept	23.26	1.30	17.95	< 0.0001
Age	−0.05	0.04	−1.48	0.1382
Gender				
Male	0.55	0.23	2.36	**0.0186**
Female	0.00	0.00	–	–
Education level				
High school or below	−1.19	0.41	−2.86	**0.0044**
Vocational school	−0.75	0.22	−3.35	**0.0009**
Bachelor degree or higher	0.00	0.00	–	–
Marital status				
Married	−0.003	0.40	− 0.01	0.9926
Unmarried/widow/divorced	0.00	0.00	–	–
Work tenure (years)	−0.02	0.03	−0.76	0.4497
Contract status				
Permanent	−0.67	0.23	−2.86	**0.0043**
Temporary	0.00	0.00	–	–
Job title				
Elementary or less	−0.22	0.24	−0.89	0.3747
Intermediate or higher	0.00	0.00	–	–
Manager responsibility				
Yes	0.19	0.23	0.82	0.4149
No	0.00	0.00	–	–
Average monthly income (RMB Yuan)				
< 2500	−0.29	0.30	−0.96	0.3386
2500~	−0.37	0.27	−1.39	0.1659
3500+	0.00	0.00	–	–
Practice setting				
CHC	−0.37	0.23	−1.61	0.1074
THC	0.00	0.00	–	–
Overall job satisfaction	−0.18	0.02	−10.25	**< 0.0001**
Emotion exhaustion	0.05	0.01	4.45	**< 0.0001**
Depersonalization	−0.01	0.03	−0.52	0.6017
Reduced personal accomplishment	−0.01	0.01	−1.22	0.2218
Workplace violence	0.12	0.04	3.45	**0.0006**

R^2^ = 0.28; F = 20.58, *P* < 0.0001

Abbreviations: *CHC* community health center, *THC* township health center

## Discussion

Promoting the building of grading healthcare system was the most important task of Chinese healthcare reforms. Based on the context of strengthening the primary health institutions was the core of healthcare system in China, assessing the turnover intention and relevant determinants among GPs not only solved the stability of human resources of primary health institutions, but also played a vital role for improving the primary healthcare.

This study investigated the turnover intention and relevant determinants among GPs in Hubei, China. Overall, the study showed that 78.35% Chinese GPs tended to have a moderate to high level of turnover intention. Our findings suggest that the significant factors associated with intention to leave were gender, education level, contract status, overall job satisfaction, EE level, and exposure to workplace violence. In addition, over 60% of GPs had ever been exposure to any type of workplace violence, and the most prevalent violence was verbal abuse (54.38%), followed by threat (33.79%), verbal sexual harassment (22.66%), physical assault (18.92%), and physical sexual harassment (7.59%).

Intriguingly, our finding was inconsistent with this result of the study by Zou et al. [41] in 2015, which showed that GPs were more likely to intend to stay in their current job than physicians. Possible reasons for this discrepancy included a relatively small size (163 GPs), and lacked a standard scale to assess the intention to stay, which may overestimate the effect size. Additionally, the study did not fully investigate other associated factors (such as job satisfaction, burnout and workplace violence) except for socio-demographic characteristics and health status.

One possible interpretation of the high level turnover intention among Chinese GPs was the heavy workload combined with a workforce shortage. In 2011, the Annual Report on the Development of Chinese Health Resources showed that the number of working hours was 53.4 h per week [42], which was obviously higher than the provisions of 44 h per week established by the Chinese labor law. In addition, a previous study showed that the harder workload could make physicians suffering from prolonged fatigue [43]. In China, the number of GPs has increased over the past few years, but it remains far shorter than what is needed. By the end of 2013, the number of GPs per 10,000 population was 1.07 [44], which was much lower than the level of developed countries such as the UK (7.2), Greece (3.1), the United States (9.6), Canada (10.4), the Netherlands (6) [45], Norway (8.2), New Zealand (7.6) and Australia (11.09) [46–48]. Another possible explanation was workplace violence, which frequently occurred within healthcare institutions, and the doctor-patient relationship was often unsatisfactory [49]. This finding showed that 60.93% of GPs was dissatisfied with doctor-patient relationships. In addition, we founded that GPs who were exposure to the workplace violence were more unsatisfactory with doctor-patient relationship than their counterparts (72.21% vs. 27.79%, *P* < 0.0001). A previous study showed that approximately 50% of health professionals felt bad in terms of relationships with patients [38], and in our study, about 63% of GPs experienced at least one type of workplace violence in the past 12 months. Third, Chinese GPs who worked in primary healthcare institutions usually had a relatively low income and educational level than those who worked in the secondary or tertiary hospital in the Chinese healthcare system. Notably, Chinese GPs were paid within a reference range set by the government though the range may vary depending on the area and their work tenure. They were more likely to leave their post and to pursue a higher general practice. Intriguingly, in the multivariable linear analysis, there was no statistically significant association between the average monthly income and turnover intention among GPs. This requires further investigation at the general practice level. The findings showed that reasonably assigned the workload and improved the income of GPs were two significant measures to ensure that GPs serve as “gatekeepers” in the primary health intuitions of Chinese healthcare system.

There was a significant difference in turnover intention between men and women GPs. We found that the likelihood of intention to leave was much greater for men than for women. One possible explanation to this finding was that men generally experienced higher levels of job dissatisfaction than women accords with previous research [23, 50]. The job satisfaction was negatively associated with turnover intention, as we found in the multivariable linear regression analysis. On the other hand, a previous investigation showed that male GPs were affected more by the work related aspects of the job (practice administration, job demands), while female GPs were affected more by the job interfering with their family life [50], which may reflect the fact that women GPs are more likely to work part time than their male colleagues. This might reflect the finding that male GPs are more likely to intention to leave than their female colleagues.

The association between exposure to workplace violence and the turnover intention among GPs was significant. GPs who had been exposed to higher frequency of workplace violence were found to have higher turnover intention compared to those who had never or less been exposed to it over a 12-month period. Previous studies showed that workplace violence was a strong predictor of workforce participation intentions [51]. In our study, approximately 63% of GPs had been exposed to at least one type of workplace violence in the past year, of which emotional abuse (EA) was the most prevalent type among GPs. As a serious occupational hazard in the hospital setting, workplace violence could contribute to various adverse consequences for the healthcare professionals, including depression, stress, burnout, sleep disruption, and job dissatisfaction [52–55]. Therefore, improving the relationship between patients and GPs is a critical challenge faced by China that warrants the increasing concern of healthcare managers and researchers. Concerted efforts being undertaken to prevent and minimize the likelihood and consequences of workplace violence in medical practice settings are essential.

Additionally, higher level of EE was also important predictor for turnover intention among GPs in our multivariable linear analysis, which was consistent with previous study [56]. Of note, in our study, we did not observe the statistically significant association between another two burnout sub-scales and turnover intention among GPs. More Chinese studies on investigating the factors associated with EE are needed, which will help to provide important clue for effective interventions to reduce the intention to leave.

There are several strengths in the present study we want to emphasize. Firstly, to date, this is the first investigation on GPs’ turnover intention and the influencing factors in China. Secondly, the large sample size significantly increased statistical power to detect social determinates of GPs’ turnover intention. It will play an important role in focusing on strategies to enhance retention as part of the wider range of initiatives for increasing the stability of GPs in primary health institutions.

Some limitations in the current study should be noted. Firstly, since this was a cross-sectional study, the causal-effect was not able to be determined. Longitudinal studies should be considered in further studies. Secondly, all information was collected from a self-reported questionnaire and the response bias was therefore unavoidable. Thirdly, the results need to be treated cautiously as GPs’ intentions to leave may not translate into action. However, other research has shown that there is a strong association between intention to leave and actually leaving [57]. Fourthly, the potential influencing factors of GPs’ turnover intention are possibly more than those we investigate; we thus failed to identify all of them. Finally, our sample was from one province of China, and thus, the generalizability of our data to other GPs in China may be limited. More studies are needed to enlarge the sample selection and include more potential factors (such as other work-related factors and occupational health factors) affecting GPs’ intention to leave their jobs, especially those factors for which specific interventions can be devised to reduce the turnover intention.

## Conclusions

In summary, this study has provided us with some understanding of Chinese GPs’ turnover intention and its influencing factors. The turnover intention among GPs in Hubei of China is high, especially in male GPs, GPs with higher education level, with temporary contact status, with lower job satisfaction, with higher levels of EE, and with more frequent exposure to workplace violence. The most prevalent violence was verbal abuse, followed by threat, verbal sexual harassment, physical assault, and physical sexual harassment. These findings could inform healthcare sector and human resource managers about the need to develop concrete strategies for retaining the workforce in general practice.

## Additional file

Additional file 1: The prevalence of different types of workplace violence. (DOCX 15 kb)

## Abbreviations

CHC: Community health center
CHS: Community health service
DP: Depersonalization
EE: Emotional exhaustion
GP: General practitioner
OR: Odds ratio
PA: Personal achievement
PHC: Primary health care
THC: Township health center
