# Supplementary material for: Turnover intention and related factors among general practitioners in Hubei, China: a cross-sectional study
Source: BMC Fam Pract. 2018 May 24;19:74. doi: 10.1186/s12875-018-0752-3 (PMC5968612; doi:10.1186/s12875-018-0752-3)
Supplement: Supplementary file 1 — The prevalence of different types of workplace violence. (DOCX 15 kb) [file 12875_2018_752_MOESM1_ESM.docx]

**Additional Table 1.The prevalence of different types of workplace violence^∗^**

| **Items** |  | **N (%)** |
| --- | --- | --- |
| **Frequency of physical assault (times/year)^§^** |  |  |
| None |  | 823 (81.08) |
| 1 |  | 111 (10.54) |
| 2-3 |  | 61 (6.01) |
| > 3 |  | 20 (1.97) |
| **Frequency of verbal abuse** **(times/year)^§^** |  |  |
| None |  | 463 (45.62) |
| 1 |  | 245 (24.14) |
| 2-3 |  | 189 (18.62) |
| > 3 |  | 118 (11.63) |
| **Frequency of threat(times/year)^§^** |  |  |
| None |  | 672 (66.21) |
| 1 |  | 195 (19.21) |
| 2-3 |  | 82 (8.08) |
| > 3 |  | 66 (6.50) |
| **Frequency of verbal sexual harassment(times/year)^§^** |  |  |
| None |  | 785 (77.34) |
| 1 |  | 114 (11.23) |
| 2-3 |  | 60 (5.91) |
| > 3 |  | 56 (5.52) |
| **Frequency of physical sexual harassment (times/year)^§^** |  |  |
| None |  | 938 (92.41) |
| 1 |  | 36 (3.55) |
| 2-3 |  | 21 (2.07) |
| > 3 |  | 20 (1.97) |

**^∗^**Respondents could choose more than one type of violence.

**^§^**Percentage of participant with missing data on workplace violence (0.10%).
